# A Novel Biosensor for the Early Detection of *Aethina tumida* via *Kodamaea ohmeri* in Honeybee Colonies

**DOI:** 10.3390/insects16040363

**Published:** 2025-04-01

**Authors:** Paola Ghisellini, Patrizia Garbati, Marco Pietropaoli, Antonella Cersini, Gabriele Pietrella, Cristina Rando, Luca Giacomelli, Stefano Ottoboni, Giovanni Formato, Roberto Eggenhöffner

**Affiliations:** 1 Department of Surgical Sciences and Integrated Diagnostics (DISC), University of Genova, Corso Europa 30, 16132 Genova, Italy; paola.ghisellini@unige.it (P.G.); p.r.garbati@gmail.com (P.G.); cristina.rando@unige.it (C.R.); luca.giacomelli@polistudium.it (L.G.); stefano.ottoboni@unige.it (S.O.); 2 Istituto Zooprofilattico Sperimentale del Lazio e della Toscana “M. Aleandri”, Via Appia Nuova 1411, 00178 Rome, Italy; marco.pietropaoli@izslt.it (M.P.); antonella.cersini@izslt.it (A.C.); gabriele.pietrella@izslt.it (G.P.); giovanni.formato@izslt.it (G.F.)

**Keywords:** *Aethina tumida* detection, biosensors, quartz crystal microbalance, *Apis mellifera* health, *Kodamaea ohmeri* detection

## Abstract

*Aethina tumida*, commonly known as the small hive beetle, poses a threat to honeybee populations, particularly *Apis mellifera*, across several European regions. Originating in sub-Saharan Africa, there is a risk of the infestation spreading from Calabria, a region in the south of Italy. The essential role played by *Apis* spp. in pollination biodiversity preservation, agricultural productivity, and the overall economy is related to the dangers posed by the invasion of *Aethina tumida*. Current detection methods often fail to identify infestations early, leading to significant colony losses. We focused on creating a biosensor to improve the detection of *Kodamaea ohmeri*, a recently identified yeast that coexists symbiotically with *Aethina tumida*. The biosensor was designed to exploit the highly sensitive quartz crystal microbalance to identify a specific peptide linked to *Kodamaea ohmeri* in honey specimens. Its gold-plated surface over quartz was functionalized with an antibody effective in recognizing the peptide associated with *Kodamaea ohmeri*, a potential warning for detecting *Aethina tumida*. Preliminary results support the possibility of using such biosensor technology to detect infestation and enhance colony management techniques for honeybees, enabling beekeepers to implement prompt and focused treatments. In addition, reducing the size and cost of these biosensors and offering user training would be very helpful in having them used in beekeeping.

## Simple Summary

This work presents a biosensor for the early detection of the small hive beetle *Aethina tumida* in honeybee colonies. The biosensor targets the symbiotic yeast *Kodamaea ohmeri*, associated with the beetle, which has been infesting hives and killing *Apis mellifera*. Based on QCM technology, this sensor detects, in honey samples, a peptide from *Kodamaea ohmeri*. It is a highly specific and sensitive device suitable for field tests to identify the infestation. Further studies are foreseen to improve the thresholds and robustness of this detection in an effort to protect sustainable agriculture and biodiversity.

## 1. Introduction

The protection of *Apis mellifera* populations is a priority, since they are highly instrumental in maintaining ecological balance and agricultural yield. These very important pollinators are, at the same time, facing serious threats in different regions [1], one of which is the Italian region of Calabria, mainly due to the infestation by *Aethina tumida*, also known as the small hive beetle (SHB) [2]. Since European honeybees do not have evolved defense mechanisms against SHB, like those from Africa, this pest is recognized as a serious threat to the viability of honeybee colonies [3]. One of the critical factors exacerbating this threat is the interaction between SHB and honeybee alarm pheromones within European honeybee colonies [4]. In addition to helping honeybees defend themselves, these pheromones attract beetles and facilitate their infestation. This situation is further aggravated by the presence of *Kodamaea ohmeri* (Ko) yeast, which is vectored by SHB and produces volatile compounds similar to alarm pheromones when grown on pollen within the hives. This not only attracts more beetles but also significantly disrupts the dynamics of the hive, leading to stress among the honeybees and, ultimately, their displacement from the hive [5]. Given the complex interplay of biological factors, managing the threat posed by SHB requires a multifaceted approach [6]. A critical challenge in this effort is the lack of efficient early detection tools. Traditional approaches using visual inspections or molecular methods are normally either insensitive, too costly, or cannot be employed in the field. Although molecular techniques are accurate, they are labor-intensive and usually unsuitable for routine monitoring by beekeepers. The proposed biosensor represents a novel approach, coupling quartz crystal microbalance technology with peptide-based specificity for Ko, a reliable biomarker for SHB infestations. This advancement addresses the limitations of existing tools, offering a scalable solution for early detection and potentially transforming beekeeping practices to improve resilience against colony losses.

However, effective management strategies also require a deep understanding of the biology of the beetle, particularly its lifecycle within the hive environment, where females lay eggs that give rise to larvae feeding on honey, pollen, and bee broods. This feeding behavior not only depletes the hive’s resources but also contributes to honey spoilage through fermentation, further worsened by the structural damage to the comb caused by larval feeding activities. Moreover, the intricate association of SHB with Ko yeast adds another layer of complexity to the infestation dynamics. This yeast plays a crucial role in the fermentation process of honey within infested hives and has been detected in various developmental stages of SHB, indicating a symbiotic relationship that influences the pest’s lifecycle and infestation patterns.

The detection of Ko yeast in association with SHB infestations [7] can be achieved through molecular techniques such as the PCR amplification of the ribosomal RNA gene (*26S rRNA*) for Ko and real-time PCR amplification of cytochrome oxidase for SHB. Reliable methods for isolating and identifying Ko from honey and hive samples were established in prior studies and protocols. The development of biosensors that use Ko’s presence as an early indicator of SHB infestations has been made possible largely due to these molecular discoveries [8]. The purpose of this work is to show that a biosensor based on quartz microbalance (QCM) can detect Ko, which may be used as a sign of an infestation of SHB. In order to evaluate its utility as an early detection tool, further verification is necessary. To successfully protect honeybee health, ecosystems, and agricultural systems, such an approach should incorporate biological insights, mechanical control methods, chemical treatments, and regulatory laws [9].

## 2. Materials and Methods

### 2.1. Sample Collection, DNA Extraction, and PCR Amplification of Kodamaea ohmeri and RT-PCR of Aethina tumida

**Figure 1:**
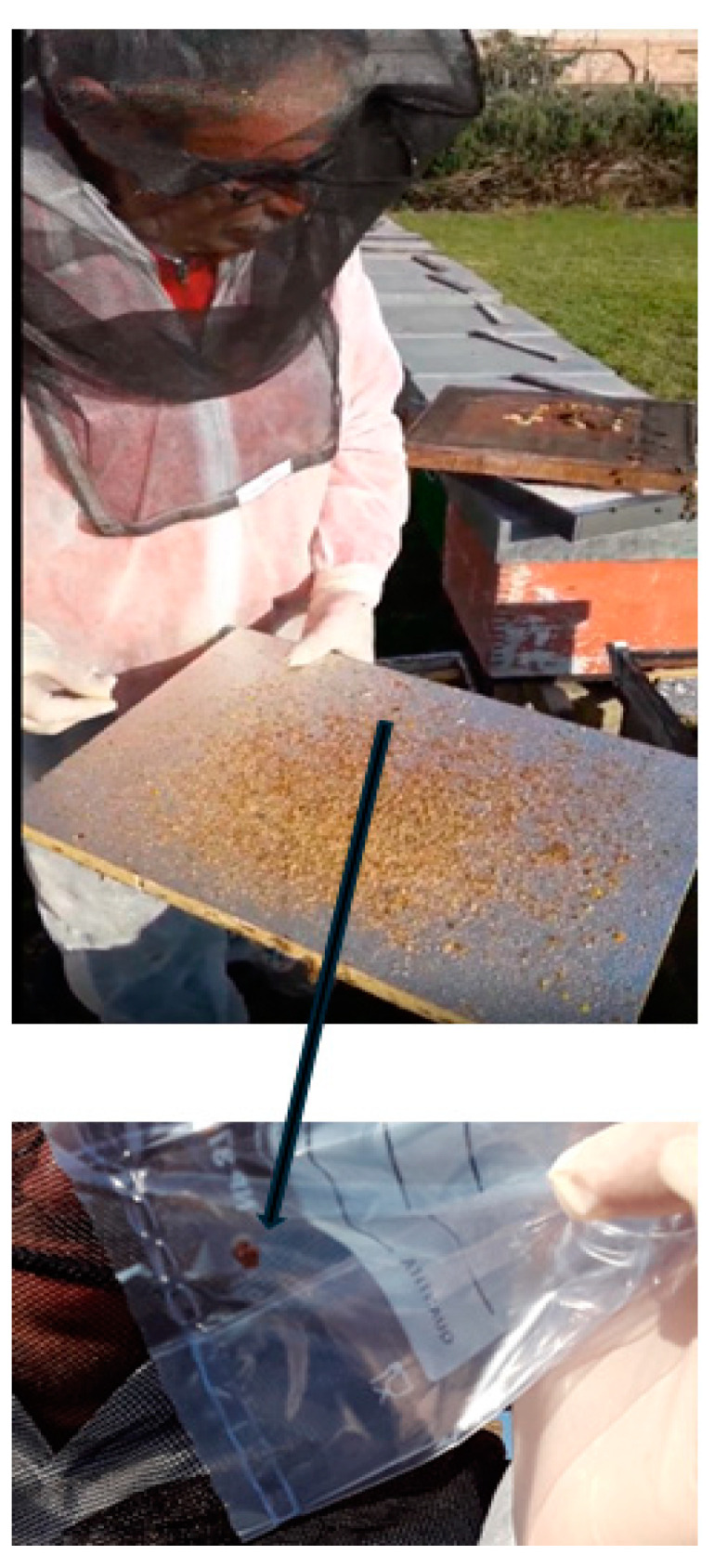
Sample collection (**upper panel**) and details of *Aethina tumida* in the (**lower panel**).

An on-site inspection was conducted following a report of infestation from a beekeeper, during which visual evidence of SHB presence was obtained as shown in Figure 1.

The honey samples were collected from hives located in the red zone of Reggio Calabria, Italy. Honey samples were collected from infested and non-infested hives, and the DNA was extracted following the standard protocol reported [10] and using the QIAamp DNA Blood Mini Kit (QIAGEN, Hilden, Germany). Finally, it was diluted in 100 µL of a 10 mM Tris-Cl, pH 8,5 buffer, and stored at −20 °C until further use. Several tests were conducted to validate the presence of Ko and SHB in different hive components such as honey debris and comb for the subsequent molecular analyses, following the above protocols.

The PCR amplification of Ko was performed using the specific primers reported in Table 1 for the *26S rRNA* gene region of Ko [11], targeting a 320 bp fragment. The amplification conditions included the following: denaturation at 95 °C for 10 min, 40 cycles made up of 94 °C for 30 s, annealing at 53 °C for 30 s and 72 °C for 1 min, and elongation at 72 °C for 1 min. PCR products were verified via gel electrophoresis and visualized under UV light.

For SHB detection, the primer pair and probe for real-time PCR were selected from the literature and reported in Table 1. They are specific to SHB, since the comparison with the 15 sequences coding for Cytochrome Oxidase I (COI) available in GenBank. It was verified in silico that these sequences do not share homology with the COI of *Apis mellifera* L., the COI of the wax moth (*Galleria mellonella*) commonly found in apiaries, the COI of *Varroa destructor* (Acari: Varroidae), and the COI of *Tropilaelaps* spp. (Acari: Laelapidae). These primers and probe amplify a 109 bp fragment under the amplification conditions reported in the literature [12].

### 2.2. Identification of a Peptide from Kodamaea ohmeri

A specific peptide indicative of the presence of Ko was identified via the PCR analysis of ribosomal RNA sequences from contaminated honey [10]. In detail, the peptide sequence was obtained from the sequencing of the 300 bp amplicon related to the ITS2 of Ko isolated in 2015 from the SHB outbreak in the province of Reggio Calabria, Italy.

The nucleotide sequence of peptide was converted to a peptide sequence using the program In-Silicon Online (http://insilico.net/tools/biology/sequence_conversion, accessed on 25 March 2025). This peptide was synthesized by SynPeptide Co., Ltd. (Shanghai, China) and used to produce specific polyclonal antibodies (antipeptides) with Twin Helix (RHO-Milan, Italy).

### 2.3. Development of a Micro/Nanogravimetric Biosensor for Detecting Kodamaea ohmeri Contamination

A QCM Wi2 instrument from Novaetech S.R.L. (Pompei, Napoli, Italy) was selected for its compactness, versatility, and suitability for on-field applications. It is designed to accommodate gold-plated electrodes with a front electrode with a diameter of 11.20 mm, a working frequency of 5 MHz, and a sensitivity of 0.017 µg/Hz. This speaks of the QCM’s ability to identify biomolecules since it can cover a wide range of sensitivities, which is essential for the precise measurements needed in advanced biosensing applications. The theoretical foundation of the operation of the QCM is grounded in the Sauerbrey equation, which correlates mass changes to frequency shifts, assuming that the deposited mass is rigidly anchored and evenly distributed across the electrode surface.

(1) $$\Delta f=-\frac{2f_{0}^{2}}{A\sqrt{\rho_{q}\mu_{q}}}\Delta m$$

where *f*_0_ is the resonance frequency (5 MHz in our case), Δ*f*_0_ is the frequency change (Hz), Δ*m* is the mass change (g), *A* is the active crystal area (i.e., the deposition electrode area, cm^2^), *ρ_q_* = 2.648 g/cm^3^ is the quartz density, and *µ_q_* = 2.947 × 10^11^ g·cm^−1^·s^−2^ (i.e., poise) is the AT-cut quartz shear modulus.

### 2.4. Surface Immobilization Protocol

A His-tagged protein A mutant derived from *Staphylococcus aureus* featured a 6xHis tag at its N-terminus and 5 fragment crystallizable (Fc)-specific binding sites, while nonspecific adsorption sites were deleted [13]. This protein, known for its high binding affinity for Fc fragments and expressed in the bacterial outer membrane, was supplied by BioVision Abcam, Cambridge, CB2 0AX, UK. A protein solution at a concentration of 1 mg/mL was prepared in 150 mM phosphate-buffered saline (PBS) by Sigma–Aldrich/Merck Life Science S.r.L., Milan, Italy. A 200 µL aliquot of protein solution was applied to the gold QCM electrode. The high affinity of the 6xHis tag for gold facilitated the formation of a monolayer on the sensor surface, ensuring the precise molecular orientation of the protein. Following a two-hour incubation at room temperature to allow protein adhesion, the sample was rinsed with distilled water to remove any unbound material, followed by air-drying. Using a protocol akin to that used for protein A deposition, an antipeptide originating from a 1.2 mg/mL stock solution in 5 mM PBS was applied. We conducted frequency measurements using the QCM to verify the successful coating of the gold surface of the quartz crystal with a stable layer of protein A linked to the antipeptide [14], as discussed in Section 3.3.

### 2.5. Peptide Detection

For each assessment, 200 µL of a 0.125 mg/mL peptide solution in 5 mM PBS was deposited onto the functionalized quartz crystals. The choice of this dosage was made considering earlier research that found comparable peptide levels in hives afflicted with SHB. To properly evaluate the biosensor’s sensitivity, further research will examine how it reacts to a larger range of concentrations.

These samples were then incubated at 4 °C overnight. The solution on the quartz surface was subsequently removed by allowing it to slide off. Washing procedures, as described above, were performed, and the quartz crystals were allowed to air-dry at room temperature. We used a control solution consisting only of PBS at the same concentration (5 mM) as the previous solution.

## 3. Results and Discussions

### 3.1. Detection of Kodamaea ohmeri and Aethina tumida in Hive Samples

Quantitative real-time PCR was used to detect SHB, while qualitative PCR was employed for Ko. A total of 15 samples were collected from hives, including honeycomb and hive bottom, in the SHB outbreak zone in Reggio Calabria, Italy, tested for the presence of SHB and Ko as reported in Table 2. The 15 samples tested positive for both SHB and Ko, excluding 2 RT-PCR invalid findings (sample ID 10 and 14). This supports the hypothesis that Ko could serve as an indicator of hive health, strongly indicating its specificity as a specific marker for SHB infestations.

### 3.2. Molecular Detection of Kodamaea ohmeri in Hive Samples

**Figure 2:**
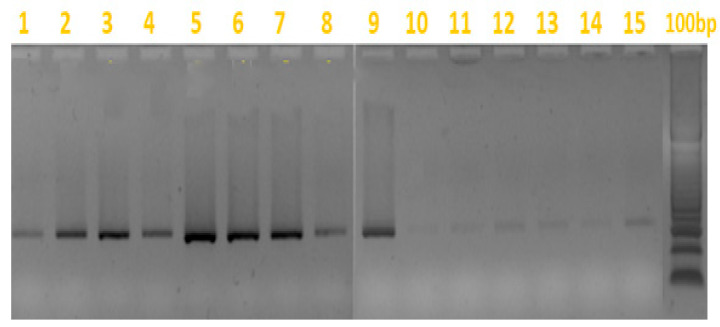
Gel electrophoresis of PCR products for *Kodamaea ohmeri*. Gel electrophoresis showing the PCR amplification of the ITS2 region of *Aethina tumida* infestation. Lanes 1–15 represent different hive samples, as follows: all samples were tested positive, samples 1 to 9 were taken from honeycomb swabs (the same samples as in Table 3) and positivity is particularly evident; samples 10 to 15 were also positive but less visible, and these were collected from hive bottom swabs. The molecular weight markers 100 bp plus the DNA ladder on the right indicate fragment sizes of 1500–100 bp.

In Figure 2, the gel electrophoresis results show the successful PCR amplification of the internal transcribed spacer (ITS2) region of Ko from hive swab samples. Positive PCR results are particularly visible in lanes 2, 3, 5, 6, 7, and 9, showing clear bands around 300 bp, confirming the presence of Ko, as reported in Table 2.

Sequencing analysis of PCR products in Table 3 yielded a 97–99% match to the *Ko 26S rRNA* gene (Accession Number MG367286.1). The sequence was detected to correspond to the KBPAP56 internal transcribed spacer 2 of the Ko strain KBPAP56 and to a partial sequence of the ribosomal RNA gene subunit.

### 3.3. Peptide and Anti-Peptide Productions

The above nucleotide sequence of MG367286.1 was converted to the following peptide sequence: ESRAARPPAPYKALSTSRVVWECSSKWVVNSI. This peptide was synthesized and used to produce specific polyclonal antibodies (antipeptides). This antipeptide was subsequently used to functionalize the QCM quartz crystal gold surface, creating a highly specific biosensor for the detection of Ko in honey samples. Further, the use of a peptide biomarker represents a novel application within the context of biosensor technology, marking an advancement over previously reported methods that lack specificity or require laboratory-based analyses.

### 3.4. Validation of Surface Immobilization

The first step, the successful deposition of the protein A layer, was confirmed by a decrease in the resonance frequency of the crystal, which ranged between 900 and 2500 Hz, indicating a mass increase in the three repeated experiments with 5 MHz crystals. Using a protocol akin to that used for protein A deposition, 200 µL of a stock solution of the antipeptide was applied. The successful deposition of the antipeptide was also confirmed by the decrease in the resonance frequency of the crystal, which ranged between 780 and 4500 Hz, indicating an increase in mass for the stable anchoring of the antipeptide.

### 3.5. Detection of Kodamaea ohmeri from the Biosensor

The peptide selected for this study was identified through a molecular biology study on various SHB-infested honey and hive product samples; details are reported in Section 3.1. This analysis validated the peptide identified as a representative biomarker of SHB infestation. The QCM instrument measures the frequency shift after peptide deposition on the antipeptide-functionalized quartz, indicating that a mass increase is attributable to the formation of the antipeptide–peptide complex. Approximately ten to hundreds of micrograms of peptide were detected, confirming the successful design of the strategy. Furthermore, the specific adhesion of the target substance to the substrate at the end of the rinsing procedure was established, thereby confirming the biosensor’s ability to precisely identify Ko in honey samples. The control samples treated with PBS alone exhibited negligible frequency variations, confirming that the significant changes observed in the peptide-treated samples are due to the specific recognition of the biological entities of analytical interest.

**Figure 3:**
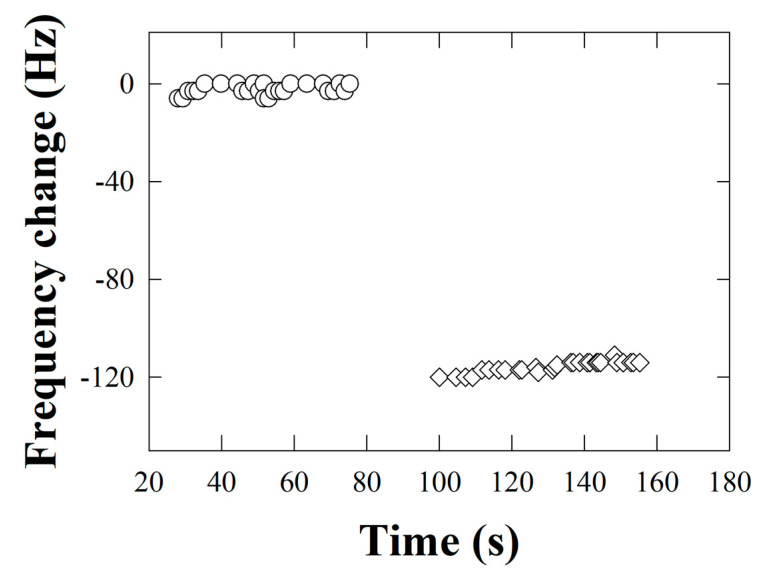
Changes in the frequency of oscillation of the quartz crystal correspond to the formation of the antipeptide–peptide complex. Open circles: frequency measurements of the functionalized quartz crystal before the addition of the peptide, taken as a zero reference to report the frequency shift. Open diamonds: measurements after peptide capture on the crystal quartz sensor.

The pivotal outcome of this methodology is depicted in Figure 3, which illustrates the frequency drop over time, a reduction attributed to the accumulation of mass on the biosensor surface due to the successful detection of the target peptide from Ko.

Control experiments conducted under identical conditions, but in the absence of the target peptide using PBS alone, demonstrated negligible frequency shifts, as evidenced by the stability of the data points in the control set within Figure 3. The suggested method’s effectiveness in identifying the presence of the Ko-associated peptide was demonstrated by the notable drop in frequency seen in the experimental set that was exposed to the peptide solution.

In essence, Figure 3 validates the utility of the developed microgravimetric biosensor technique for identifying the Ko peptide. The observed frequency reduction reflects the increase in mass deposition on the biosensor due to the captured peptide, thereby corroborating the effectiveness of the peptide-specific antibody in biosensor design.

Our biosensor utilizes QCM technology, which is effective at biomolecule detection due to its sensitivity to minute mass variations. This concept is employed to detect a specific peptide originating from the yeast Ko, serving as an early warning indicator for potential SHB infestation. The goal was to achieve excellent specificity for the detection of Ko. Since Ko is often found in SHB-infested hives, a sensor that detects its presence can be useful as a prevention system and should be seen as part of a broader toolkit for hive monitoring. While the present research does not focus on food safety evaluations, should future assessments determine that *K. ohmeri* in honey poses a food safety concern, the biosensor could be utilized for this distinct purpose. In this instance, additional regulations would be necessary for the proper implementation of the biosensor.

## 4. Conclusions

This research introduces an advanced biosensor for honeybee health monitoring, specifically designed to detect the Ko yeast as an indicator of SHB. It is highly precise, portable, and possesses significant potential for practical application in the field. Additional research is required to enhance sensitivity, elevate early detection thresholds, optimize robustness against varying environmental conditions, and minimize the costs of these devices for rapid dissemination. The integration of the current biosensor into standard practice could enable stakeholders, including beekeepers, honey producers, and local governments, to proactively combat SHB, thereby enhancing the resilience of pollination systems and ultimately safeguarding agricultural productivity and biodiversity.

The development of biosensors facilitated the acquisition of a method with high specificity through the meticulous selection of the target peptide, the immobilization strategy, and the application of QCM technology. The QCM-based biosensor is a viable alternative that requires additional research for optimization before implementation. Subsequent studies must focus on the enhancement of the sensor’s sensitivity, thereby reinforcing its efficacy in early detection. The incorporation of this approach into beekeeping practices can facilitate proactive colony management and may mitigate the detrimental effects of delayed alerts regarding SHB infestation.

## Figures and Tables

**Table 1:** Sequences of the primers for the PCR of *Kodamaea ohmeri* and the specific primers and probe for RT-PCR of *Aethina tumida*.

Ko Primers for PCR	Sequence
Ko Fw	5′-TAATTTGAAGATTGCGTCTTG-3′
Ko Rv	5′-TACCCACACTGACAATCTGAC-3′
**SHB Primer/Probe name for RT-PCR**	**Sequence**
Fw SBH207F	5′-TCTAAATACTACTTTCTTCGACCCAT(A/G)-3′
Rv SBH315R 5′	5′-TCCTGGTAGAATTAAAATATAAACTTCTGG-3′
SBH245T PROBE	5′-FAM-ATCCAATCCTATACCAACACTTATTTTGATTCTTCGGAC-3′-TAMRA

**Table 2:** Real-time PCR detection of *Aethina tumida* and PCR of *Kodamaea ohmeri* in 15 samples (all honey samples, except 13, which was a honeycomb sample).

Sample ID	Real-Time PCR for *Aethina tumida* (Ct Value)	Qualitative PCR for *Kodamaea ohmeri* (Positive/Negative)
1	31.23	Positive
2	32.01	Positive
3	31.55	Positive
4	30.47	Positive
5	29	Positive
6	31.4	Positive
7	30.72	Positive
8	31.95	Positive
9	31.22	Positive
10	0	Positive
11	35	Positive
12	33.92	Positive
13	32.49	Positive
14	0	Positive
15	27.86	Positive

**Table 3:** Sequencing results in honeycomb swab samples testing positive for *Kodamaea ohmeri*.

Sample ID	Query Cover (%)	Identity (%)
1	99%	97%
2	96%	99%
3	96%	99%
4	97%	99%
5	99%	98%
7	96%	99%
8	94%	99%
9	97%	99%

## Data Availability

The original contributions presented in this study are included in the article. Further inquiries can be directed to the corresponding author.

## Abbreviations

The following abbreviations are used in this manuscript:

SHB	Small Hive Beetle, *Aethina tumida*
Ko	*Kodamaea ohmeri*
QCM	Quartz Crystal Microbalance
COI	Cytochrome Oxidase I
ITS2	Internal Transcribed Spacer
